# Analysis of Serum MicroRNA-122 Expression at Different Stages of Chronic Hepatitis B Virus Infection

**DOI:** 10.1155/2021/9957440

**Published:** 2021-06-03

**Authors:** Wenjun Liu, Xinxiang He, Fei Huang

**Affiliations:** Department of Infectious Diseases, Jingzhou Central Hospital, The Second Clinical Medical College, Yangtze University, Jingzhou, Hubei 434020, China

## Abstract

**Objective:**

To investigate the expression of microRNA-122 (miR-122) in the progression of chronic hepatitis B virus- (HBV-) infected liver diseases, thus determining the role of serum miR-122 as a marker of HBV-caused liver injury.

**Methods:**

Sera were collected from patients with different stages of HBV infection (*n* = 63) and healthy volunteers (*n* = 11). And the serum miR-122 levels were detected using RT-qPCR. Moreover, an analysis was applied for identifying the specific correlation of the miR-122 level with HBV DNA, HBeAg, and ALT levels. After liver biopsy, Ishak scoring was utilized for evaluation of the fibrosis stage and the histological activity index (HAI).

**Results:**

We confirmed, in the serum, increased miR-122 expression in HBV-infected patients and its highest expression in chronic HBV carriers, based on such comparison between the healthy controls and patients. The correlation analysis results were taken as confirmation of the positive relationship of miR-122 with HBV DNA (*r* = 0.354, *P* = 0.005) and ALT (*r* = 0.331, *P* = 0.009). But no correlation of this molecule with HBeAg levels was found (*P* = 0.187). In comparison with the HBeAg-negative patients, serum miR-122 expression showed an increase in the HBeAg-positive patients (*P* = 0.001). miR-122 expression, in addition, was of a significant correlation with HAI, but not with the liver fibrosis score.

**Conclusion:**

The peak of the serum miR-122 expression normally occurs in the early stage of the progression from the HBV carrier phase to chronic hepatitis to cirrhosis. This molecule can be considered as a marker for evaluation of HBV-caused liver injury.

## 1. Introduction

Hepatitis B virus (HBV) infection is often mentioned as a serious public health issue which attacks 350-400 million people [1, 2]. In chronic hepatitis B (CHB) patients, liver cirrhosis carries a high risk to develop, as dose hepatocellular carcinoma [3]. Chronic HBV infection might go through several stages: chronic HBV carrier phase, CHB, cirrhosis, and ultimately HCC. Chronic HBV carriers can be defined as those people in the immune tolerance phase who are positive for HBsAg, HBeAg, and HBV DNA and have normal liver function and no obvious abnormalities in the liver histology, and this situation might be maintained for several years or even decades [4, 5]. Patients with CHB demonstrate abnormal liver function or/and abnormal liver histology. When patients progress to liver cirrhosis, 3% 6% patients have the possibility to develop into primary HCC [6]. Therefore, it is essential to figure out regulation factors in the progression of HBV infection-related diseases, so as to better designate targeted treatments.

MicroRNAs (miRNAs) are noncoding RNAs with a length of about 21 to 23 nt and with high evolutionary conservation [7, 8]. In 2008, miRNAs were first extracted and detected in body fluids, and afterwards, miRNAs exist in large quantities in the serum of healthy individuals and cancer patients, as well as diabetic patients according to some studies [9]. Subsequently, researchers got confirmation that miRNAs exist in human urine, saliva, amniotic fluid, and hydrothorax [5]. At present, association between several kinds of miRNAs and HBV replication has been reported. For example, miR-1 has the capability to stimulate the farnesoid X receptor, thereby increasing the activity of the HBV core promoter and affecting HBV replication [10]. miR-210 and miR-199a-3p can inhibit HBV replication as well.

Among the total hepatic miRNAs, approximately 70% are liver-specific miR-122 [11, 12]. Wang et al. pointed out that miR-122 affects both p53 and cyclin G-1 and consequently HBV replication [13]. Specifically speaking, after transfecting liver tumor cell lines with antisense nucleic acids or mimics of miR-122, the inhibition of HBV replication was found by overexpression of miR-122 and promotion of HBV replication by depletion of endogenous miR-122 [14]. After determination that miR-122 acting on downregulation of heme oxygenase-1 (HO-1) [15], further study has proven that HO-1 achieves suppression of HBV replication via functioning in the reduction of HBV core protein stability and the suppression of HBV covalently closed circular DNA (cccDNA) elongation [14].

On the basis of the above researches, we hypothesized that serum miR-122 can act to evaluate conditions HBV-induced liver injury at different stages. And there are no literature reports on the expression of miR-122 in different stages of HCC. Therefore, we analyzed the association of serum miR-122 with HBV DNA, ALT, and fibrosis degree. Furthermore, the comparison of levels of miR-122 at different stages of chronic HBV infection was carried out.

## 2. Materials and Methods

### 2.1. Sample Collection

On completion of the collection of serum from HBV-infected patients (*n* = 62) who underwent liver biopsy in the Infectious Diseases Ward of the Jingzhou Central Hospital from 2018 to 2019 and healthy volunteers (*n* = 11), the collected serum was anticoagulated with EDTA and stored at −80°C. Before serum collection, the informed consent signed by each subject was obtained. And an approval was gained from the Medical Ethics Committee of Jingzhou Central Hospital (JZCH2016006).

### 2.2. Screening Criteria

*Inclusion criteria*: (1) control group: people who are confirmed healthy by recent physical examination. (2) HBV carrier group: people who are positive for HBsAg, HBeAg, and HBV DNA with persistently normal levels of serum ALT and AST and with no obvious abnormality shown in their liver biopsy. (3) CHB group: people who once suffered from hepatitis B or who had positive HBsAg for more than 6 months and positive HBsAg and/or HBV DNA till now and had a continual or repeated increase of ALT or who were found to have hepatitis lesions in their liver biopsy. (4) Cirrhosis group: people who were diagnosed as cirrhosis belonging to the Child-Pugh A grade by clinical images and/or liver biopsy.

*Exclusion criteria*: (1) people with mixed infection of hepatitis B and C, alcoholic liver disease, fatty liver, HIV infection, drug-induced liver injury, autoimmune liver disease, or tumors. (2) People who once received or are receiving antiviral therapy.

### 2.3. Detection of HBV Indicators in Serum

The COBAS AmpliPrep/COBAS TaqMan quantitative detection system (Roche, Switzerland), with 12 IU/mL as a detection limit, was utilized for determining the content of HBV DNA in serum following the reagent instructions. Meanwhile, the concentrations of HBeAg and HBsAg were measured by chemiluminescence microparticle immunoassay. Positive criteria for HBeAg: when the ratio of sample signal to cutoff (S/CO) < 1.000, the sample is considered as nonreactive; when the S/CO value ≥ 1.000, the sample is considered as reactive. The content of ALT was measured by the reduction coenzyme method.

### 2.4. Liver Pathology

After the sectioning step of paraffin-embedded biopsied liver tissues, the staining step by hematoxylin-eosin (HE) and Masson was performed. The Ishak scoring system [16] was utilized for evaluation of the fibrosis stage and histological activity index (HAI). The HAI score is aimed at assessing the degree of necrotizing inflammation (0–6 points). The degree of fibrosis is ranged from 0 (no fibrosis) to 6 (possible or definite cirrhosis).

### 2.5. Quantitative Reverse Transcription PCR (RT-qPCR)

Total RNA was separated from serum by utilizing the mirVana PARIS Kit (AM1556, Applied Biosystems, USA). After measuring RNA concentration on a NanoDrop 2000 Nucleic Acid Analyzer (NanoDrop, USA), RNA was reversely transcribed into cDNA according to the instructions of the TaqMan MicroRNA Reverse Transcription Kit (Applied Biosystems, USA). Finally, RT-qPCR reactions were performed by the TaqMan MicroRNA Assay on a 7500HT real-time quantitative PCR instrument (Applied Biosystems, USA). The reaction was carried out with conditions including 95°C for 5 min and 40 cycles of 95°C for 15 s, 60°C for 60 s, and 72°C for 30 s. The relative expression of miR-122 was calculated using the 2^−*ΔΔ*Ct^ method with U6 as an internal reference. The information of the probe primers synthesized by ABI Company is shown in Table 1.

### 2.6. Statistical Analysis

SPSS 17.0 software was used for statistical analysis. All experimental data were expressed as mean ± standard deviation (SD). For comparison among multiple groups, Kruskal–Wallis *H* test was adopted, while Mann–Whitney *U* test for comparison between 2 groups. Correlation coefficients were detected using bivariable pairwise correlation analysis. Continuous variables were analyzed by the Pearson method, and categorical variables were analyzed by the Spearman method. *P* < 0.05 indicates that the difference is statistically significant.

## 3. Results

### 3.1. Baseline Information

There were 73 serum samples, 62 from the HBV-infected patients and 11 from the control group. Baseline information about all subjects is described in Table 2.

### 3.2. Serum miR-122 Levels at Different Stages of HBV Infection

**Figure 1:**
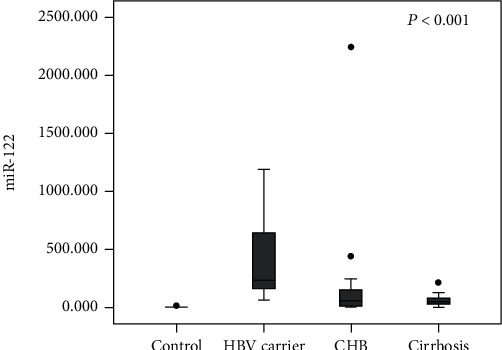
Serum miR-122 expression in each group.

RT-qPCR results indicated that in the serum, increased miR-122 expression in HBV-infected patients and its highest expression in chronic HBV carriers was confirmed based on such comparison between the healthy controls and patients. Among all the infected patients, in comparison with the CHB and cirrhosis groups, the highest miR-122 expression showed in the HBV carrier group (*P* < 0.001). No marked differences between the CHB and cirrhosis groups in the miR-122 level was identified (*P* = 0.734) (Figure 1).

### 3.3. Correlation of Serum miR-122 Expression with HBV DNA, HBeAg, and ALT

**Figure 2:**
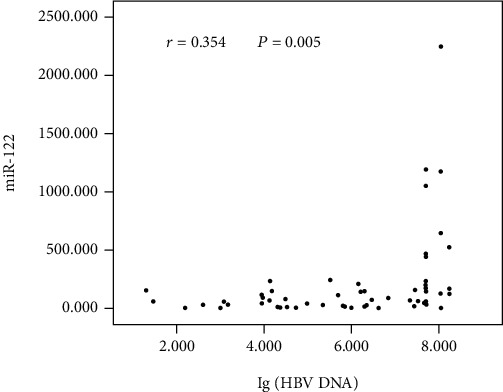
Correlation analysis between HBV DNA and miR-122 expression in serum.

**Figure 3:**
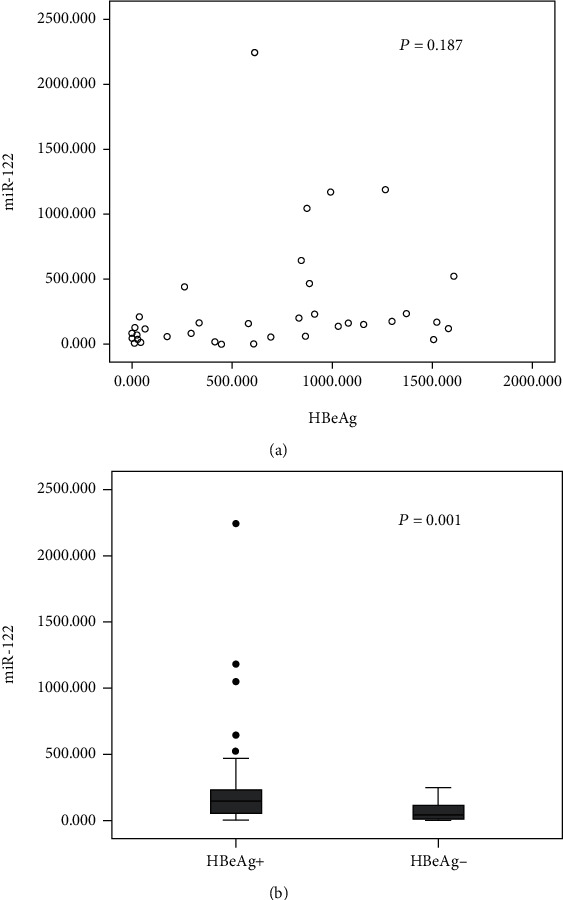
(a) Correlation between HBeAg and miR-122 expression in serum. (b) Comparison of serum miR-122 expression between the HBeAg-positive group and the HBeAg-negative group.

**Figure 4:**
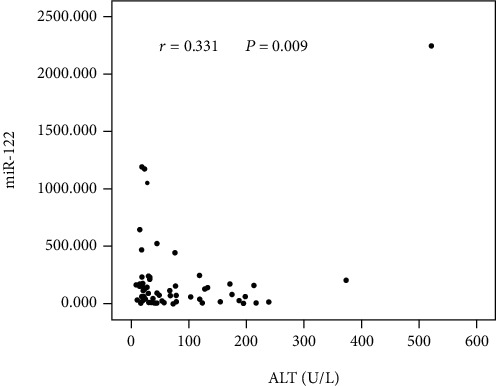
ALT level and serum miR-122 expression.

Determination of the association between virological data and miR-122 in 73 serum samples was subsequently carried out. As shown in Figure 2, in the serum, HBV DNA was increased, as was miR-122, indicating a positive correlation (*r* = 0.354, *P* = 0.005). But no significant association between HBeAg and miR-122 was identified (*P* = 0.187) (Figure 3(a)). Interestingly, in comparison with the HBeAg-negative patients (*n* = 26), serum miR-122 expression showed an elevation in the HBeAg-positive patients (*n* = 36) (*P* = 0.001) (Figure 3(b)). Additionally, the serum miR-122 level was of a positive relationship with the ALT level (*r* = 0.331, *P* = 0.009) (Figure 4).

### 3.4. Correlation of Serum miR-122 Expression with Liver Histology

**Figure 5:**
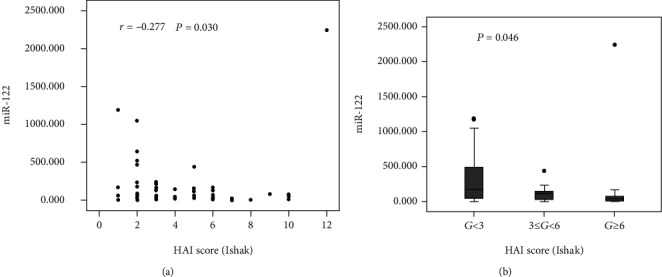
Correlation of serum miR-122 with inflammation degree. (a) Correlation analysis between HAI score (b) and miR-122 level. (b) Serum miR-122 expression in groups with different HAI scores. *G*: grading of inflammation.

**Figure 6:**
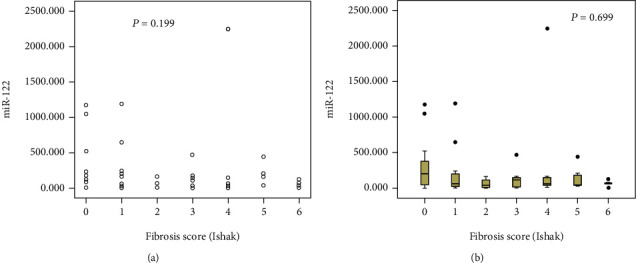
Correlation of serum miR-122 with liver fibrosis. (a) Correlation analysis between fibrosis score and miR-122 level. (b) Serum miR-122 expression in patients with different fibrosis scores.

A higher miR-122 level led to a lower HAI score, suggesting a negative relationship (*r* = −0.277, *P* = 0.030) (Figure 5(a)). Additionally, a different degree of inflammation (*G* < 3, 3 ≤ *G* < 6, *G* ≥ 6) resulted in different miR-122 levels (*P* = 0.046) (Figure 5(b)). In particular, the expression of miR-122 was the highest when *G* < 3. Furthermore, no marked association of miR-122 with the fibrosis score (*P* = 0.199) (Figure 6(a)) and degrees of fibrosis (*P* = 0.699) was revealed (Figure 6(b)).

## 4. Discussion

Association between HBV replication and miR-122 has been reported. When cells are infected with HBV, apoptotic or necrotic cells passively release miR-122 into the circulatory system. Some researchers believe that the release of miRNA into the blood may be one of the mechanisms by which cells adapt to the environment [17]. In the present study, we found that the expression of serum miR-122 was significantly higher in chronically HBV-infected patients than in healthy ones, which is consistent with the findings of several studies both at home and abroad [18–20]. Serum miRNAs always maintain a stable level, but when miRNAs are released from damaged liver cells, they continue to accumulate and reach a higher level. This can be the reason why in comparison with healthy controls, the serum miR-122 level is elevated in HBV-infected patients.

It is generally believed that in comparison with HBV carriers, the degree of liver injury and miR-122 should be higher in CHB and cirrhosis patients. However, the opposite fact in this study is that in comparison with the CHB and cirrhosis groups, miR-122 expression markedly rose in the HBV carriers; there were no marked differences between the CHB and cirrhosis groups. That is to say, the maximum expression of serum miR-122 mainly occurs in the early stage of HBV infection-induced liver disease. Adding to that, domestic researchers have revealed that the changes of miRNA expression normally are seen in the early progression which HBV infection eventually develops into HCC [21]. Therefore, we speculated that the differences of miR-122 expression in liver tissue and cells may be similar to those in HBV carriers, patients with CHB, or cirrhosis. And these differences might suggest that miR-122 may serve as one of the incentive factors in the progression of HBV-induced diseases.

HBV DNA, as the most direct and reliable indicator of viral replication, is able to directly reflect the viral levels in vivo as well as the infectivity [22]. In HBV-infected patients, the marked elevation of the serum miR-122 level has been mentioned by a couple of studies [18–20]. We revealed that miR-122 expression was of a significant correlation with HBV DNA, which is consistent with the results reported abroad [20, 23]. Beyond that, HBeAg is an immunoregulatory factor that stimulates different types of cell subsets and secretes various cytokines. What is more, HBeAg inhibits the cytotoxicity of T cells in order to form immune tolerance to HBV infection [24]. Our study suggested no correlation between HBeAg quantification and miR-122. However, in comparison with the HBeAg-negative patients, serum miR-122 expression appeared to be increased in the serum of HBeAg-positive patients, which is consistent with the results drawn by Arataki et al. [20]. These results can be taken as confirmation of a close relationship of miR-122 with HBV DNA and HBeAg content.

ALT is a biochemical marker applied widely for evaluating conditions of liver injury. In the serum samples, we herein found a positive relationship between miR-122 and ALT, which is consistent with the foreign studies [18, 23]. The gold standard for the assessment of liver inflammation degree is deemed to be liver histology. In this study, 62 chronic HBV-infected patients underwent liver biopsy. By utilizing the Ishak scoring system, the HAI and fibrosis stage were evaluated. We got results in determination that serum miR-122 has a significant relation with the HAI score, but not with the fibrosis score. Furthermore, among different degrees of fibrosis, no significant differences were found in the miR-122 expression, which is consistent with the results reported by Arataki et al. [20] and Waidmann et al. [23]. Further studies revealed that miR-122 levels would be affected when the inflammatory degree is different (*G* < 3, 3 ≤ *G* < 6, *G* ≥ 6). Collectively, serum miR-122 can serve as a potential marker for evaluation of HBV infection-induced liver injury.

## 5. Conclusion

In summary, we found that the peak of serum miR-122 levels normally occurs in the early stages of the progression from the HBV carrier stage to chronic hepatitis to cirrhosis. This provides a novel perspective and direction for mechanistic studies of HBV-induced disease. However, there were too many inadequate experimental samples in this study. More clinical samples are required to further investigate the expression of miR-122in HBV-infected liver disease.

## Figures and Tables

**Table 1:** Probes used to detect gene expression.

Name	TaqMan Assay ID	Accession number	Chromosome location
U6	00197	NR-004394^∗∗^	15q23
miR-122	002245	MIMAT0000421^∗^	18q21.31

^∗^miRBase database accession number; ^∗∗^Entrez Gene ID.

**Table 2:** Patient characteristics.

Groups	Age	HBV DNA (lg copies/mL)	ALT (U/L)
HBV carrier group (*n* = 14)	30.29 ± 11.19	7.79 ± 0.37	22.8 ± 9.28
CHB group (*n* = 34)	38.0 ± 10.81	5.39 ± 2.05	102.7 ± 111.27
Cirrhosis group (*n* = 14)	43.29 ± 6.28	5.89 ± 1.78	78.93 ± 66.76
Control group (*n* = 11)	27.45 ± 6.20		

Data are presented as mean ± SD. CHB: chronic hepatitis B.

## Data Availability

Data are available in the article.
